# Synthesis of Magnesium- and Silicon-modified Hydroxyapatites by Microwave-Assisted Method

**DOI:** 10.1038/s41598-019-50777-x

**Published:** 2019-10-16

**Authors:** Liudmila A. Rasskazova, Ilya V. Zhuk, Natalia M. Korotchenko, Anton S. Brichkov, Yu-Wen Chen, Evgeniy A. Paukshtis, Vladimir K. Ivanov, Irina A. Kurzina, Vladimir V. Kozik

**Affiliations:** 1 National Research Tomsk State University, 36 Lenina Avenue, Tomsk, 634050 Russia; 2 Department of Chemical Engineering, National Central University, Jhongli, 32001 Taiwan

**Keywords:** Biological techniques, Molecular biophysics, Chemical biology

## Abstract

Nanopowders of hydroxyapatite (HA), modified by magnesium (MgHA) and by silicon (SiHA) were obtained by liquid-phase microwave synthesis method. X-ray diffraction and IR spectroscopy results showed that Mg^2+^ and SiO_4_^4−^ ions were present in the synthesized products both as secondary phases and as part of the HA phase. Whitlockite was found in the magnesium-modified HA (MgHA) and larnite was found in the silicon-modified HA (SiHA); ion substitution for both materials resulted in solid solutions. In the synthesized samples of modified HA, the increase of particle size of powders was in the order HA < SiHA < MgHA, which was calculated through data specific surface area and measured pycnometric density of the powders. The Lewis acid sites (Ca^2+^, Mg^2+^, Si^4+^) were present using spectral probes on the surface of the samples of HA, MgHA, and SiHA, and the acidity of these sites decreased in the order SiHA > MgHA > HA. The rates of calcium phosphate layer deposition on the surface of these materials at 37 °C in the model simulated body fluid solution showed similar dependence.

## Introduction

The problem of finding material for implantation has existed since ancient times. However, towards the end of the twentieth century the requirements for such materials and an informed and scientifically grounded application began to form. In the ideal case, the material should be non-toxic, not reject the organism as a foreign body, contact with the biological system of the organism, and induce the formation of bone tissue. In addition, the implant must retain its functional properties for a certain period of time, without significant change in its structure and mechanical properties^1^.

Initially, preference was given to biologically inert - non-toxic and resistant to the biochemical effects of the organism’s materials. Such materials include corrosion-resistant metals and alloys based on steel, cobalt, chromium, and titanium. However, when functioning in the organism, metals are subjected to stretching, compression, and bending, as a result of which they are deformed. Additionally, various products are formed in the biological environment of the organism to the metal surface, adversely affecting the surrounding tissue and resulting in rejection of the implant^2^.

With the development of the chemistry of high-molecular compounds, polymeric materials have been widely used for implants: polyethylene, polyamides, polylactide, and polyglycolide, which, in addition to biological inertness, possess high mechanical properties and strength characteristics. For example, polyethylene contains residual free radicals, leading to oxidative degradation of the product^3^; polyamides undergo shrinkage and are prone to deformation and rapid loss of mechanical strength^4^; polylactide has technological difficulties in production and poor wettability^5^; polyglycolide has high rigidity and low elasticity^6^. In addition, in the hydrolysis of polylactide and polyglycolide, acids are formed, which can lead to inflammation of the surrounding tissues and subsequent rejection of the material^7^.

Significant progress has been made in the use of biologically active materials based on substances similar in chemical and phase composition to bone tissue. As a rule, this is a series of calcium phosphates. The closest in composition is hydroxyapatite (HA) (Ca_10_(PO_4_)_6_(OH)_2_), which is the main mineral component of bone tissue, enamel, and dentin. HA-based materials have many applications in medicine and materials science^8,9^. HA is used for replacement implants in the damaged bone tissue (calcium–phosphate cements, coatings, inorganic parts of composite materials)^10–14^. The crystalline structure of HA allows the substitution of elements with other elements. To modify the physicochemical and biochemical properties of HA, various ions were introduced into its structure, such as biologically active (Na^+^, Zn^2+^, CO_3_^2−^, Mg^2+^, SiO_4_^4−^) present in the natural bone tissue^15–19^, and with original HA for ions (Nd^3+^, Tb^3+^, Au^3+^, Sr^2+^, Eu^3+^) imparting new functional properties to HA structure^20–24^, thereby expanding the range of its applications in various fields.

The most important property of calcium phosphates is bioresorption, which directly depends on their solubility in the physiological conditions of the internal environment of the organism. The solubility of calcium phosphates used for reconstructing bone tissue decreases in the order α-tricalcium phosphate > tetracalcium phosphate > dicalcium phosphate > octacalcium phosphate > β-tricalcium phosphate > HА. Thus, among all the calcium phosphates, HA has the lowest resorption. Too low resorbability does not always allow the use of HA in its pure form as an implantable material. Modification of HA in various biologically active ions enables controlling the speed of its resorption, most often directed at improving HA resorption^1^.

A variety of cationic and anionic substitutions can be applied due to the flexibility of the apatite structure. The introduction of Mg^2+^ and SiO_4_^4−^ ions into the structure of HA makes it possible to control the processes of bioresorption and also facilitates the formation of bone stock on the surface of the material^25–28^. Magnesium- and silicon-modified HA show high bioactivity and resorbability, promoting the acceleration of biomineralisation and integration of implants in the area of bone defects. The resorption rate of implanted materials based on pure HA in the body environment is too low, which often leads to prolonged bone tissue fusion processes with preservation of the remains of the artificial implant. In this study, HA-based composite materials with highly resorbable secondary phases containing Са^2+^ and РO_4_^3−^ ions were used to overcome this drawback. The complexities of classical liquid-phase synthetic methods for calcium phosphates with the ratio Ca/P ≤ 1.67 are associated with the simultaneous monitoring of a large number of factors (temperature, rate of discharge of reagents, stirring of the reaction mixture, time) affecting the phase composition, homogeneity of the particles, and morphology of the product of synthesis^9^. In this study, microwave-synthesized HA powders were used to prevent these shortcomings. In microwave heating, in comparison with conventional heating, heat is generated inside the material, rather than supplied from an external heating source; therefore heating occurs immediately over the entire volume of the irradiated sample^29^.

This study investigated the crystalline phase, elemental composition, microstructure, and physicochemical and biomimetic properties of magnesium and silicon-modified hydroxyapatites that were prepared by liquid-phase microwave synthesis.

## Experimental

Liquid-phase synthesis of unmodified HA and modified MgHA and SiHA was conducted with microwave heating under the conditions described earlier^30^. Calcium nitrate tetrahydrate (Vekton, 0.5 mol/l), diammonium hydrogen phosphate (Vekton, 0.3 mol/l), aqueous ammonia (Sigma Tec, density 0.907 g/ml, ω = 25%), and chemical grade deionised water were the starting materials. The initial solutions of calcium nitrate and ammonium hydrogen phosphate were taken in volumes that provided a quantitative ratio n(Ca)/n (P) = 1,67.

The stock solutions were mixed and then exposed to 100 W microwave heating for 30 min; the pH of the reaction mixture was adjusted to ~11. Magnesium nitrate (Vekton) (0.1 mol equiv. Mg^2+^) was dissolved in a prepared calcium nitrate solution. An alcoholic solution of tetraethoxysilane (TEOS) (Sigma Aldrich) and ethyl alcohol (Sigma Tec) with a molar ratio of C_2_H_5_OH: TEOS = 1:1 was used to obtain SiHA (0.6 mol % SiO_4_^4−^)^31^. The alcoholic solution of TEOS was mixed with a prepared aqueous solution of diammonium hydrogen phosphate.

The values of molar compositions (*х* Mg^2+^ = 0.1 and *х* SiO_4_^4−^ = 0.6) corresponding to the minimum content of ions were introduced into the system to investigate the changes in the phase composition and physicochemical properties (dispersity, solubility) of HA. All the resulting powders were calcined at 800 °C for 1 h. The samples were white crystalline substances.

The X-ray diffraction analysis of НА powders was performed on the Shimadzu XRD 6000 diffractometer at Cu _Kα_ = 1.5406 Ǻ radiation at 7–55° (2Ө). The processing of the data obtained and phase identification were carried out using a database JSPDS PDF 4+ and full-profile analysis programmes POWDER CELL 2.4. The IR spectra of HA, MgHA, and SiHA powders in the KBr and BaF_2_ matrices (for correct measurement of the OH group spectra) were recorded on a Shimadzu FTIR-8300 IR-Fourier spectrometer at 4000–400 cm^−1^.

The elemental analysis and element distribution on the surface of HA, MgHA, and SiHA powders were studied with X-ray spectral microanalysis (EPMA) both for separate points of the images and for areas, using a scanning electron microscope (Leo Supra 50VP with an Oxford Instruments X-Max detector, and Zeiss Supra 55VP) and X-ray spectral microanalyser (ShiftED 300). The specific surface areas, pore volumes, and pore sizes of the powders were measured by nitrogen adsorption on a TriStar II Micromeritics device. The surface morphology of the samples was studied using scanning electron microscopes Carl Zeiss NVision 40 with 1–30 kV accelerating voltages using secondary and backscattered electron detectors. The survey was performed without preliminary deposition of conductive materials on the surface of the samples, with the Zeiss Supra 55VP (used to photograph) and JEOL-7500FA (2i0 kV accelerating voltage). The specific surface area (Ssp), volume, and pore size of the powders were measured by nitrogen adsorption followed by degassing at a pressure of ~0.1 Pa at 200 °C for 1 h on a TriStar II Micrometerics device using the BET method. A low-temperature nitrogen vapor sorption method has a relative error ∆ ± 10%.

The particle size (d) of the dispersed samples was estimated by the value specific surface area and measured pycnometric density of powders. In the ideal case, when particles of the same size have a cubic shape with an edge length l (μm), the specific surface area S_sp_ (m^2^/g) is determined by the expression S_sp_ = 6/ρ·d, where ρ (g/cm^3^) is the density of a solid, measured by liquid displacement. For real powders composed of irregularly shaped particles and of various sizes, this ratio has a more complex form, but the equation makes it possible to roughly estimate its size.

To assess the solubility of both unmodified and modified HA powders, the total calcium ion Са^2+^ concentration in physiological solution (ω(NaCl) = 0.9%) at 20 °C and 37 °C was determined by titration with Trilon B in the presence of eryochrome black T in ammonia buffer, pH 9–10. The samples were aged in physiological solution for 7 days to reach saturation with respect to the solid phase. The mean values of С_Са_^2+^concentrations (mol/l) and confidence interval (for P_*b*_ = 0.95) were calculated at least three parallel measurements.

The ability of the samples to form a calcium–phosphate layer on their surface in the model simulated body fluid (SBF) solution was evaluated by the procedure described by Rasskazova *et al*.^32^. The compressed tablets of 5 mm in diameter were placed in an SBF solution, which was identical to human plasma in its mineral composition and ion concentration. The substrates were aged in the SBF solution for 28 days at 37 °C with daily solution renewal. The rate of calcium phosphate layer (CPL) formation on the surface of substrates was assessed by the decrease in total concentration of calcium and magnesium ions (Δ*m*_(*Ca2*+ and Mg2+)_, g/l) in the SBF solution, determined by trilonometric titration. The morphology of the substrate surface with the formed calcium phosphate layer was investigated with a scanning electron microscopy (SEM) on a Hitachi TM-3000 microscope with 15 kV accelerating voltage (electron gun: 5 × 10^−2^ Pa, sample chamber: 30–50 Pa).

The acidic properties of the surface of the samples were determined by the IR spectroscopy of adsorbed CO on the surface of the pre-calcined pellets (thickness 6–12 mg/cm^2^) of HA, MgHA, SiHA, pre-calcined in vacuum at 600 °C for 1 h. Adsorption of CO was carried out at a temperature of −196,15 °C and increase in pressure of CO from 0.1 to 10 mmHg.

## Results and Discussion

### Qualitative composition

**Figure 1:**
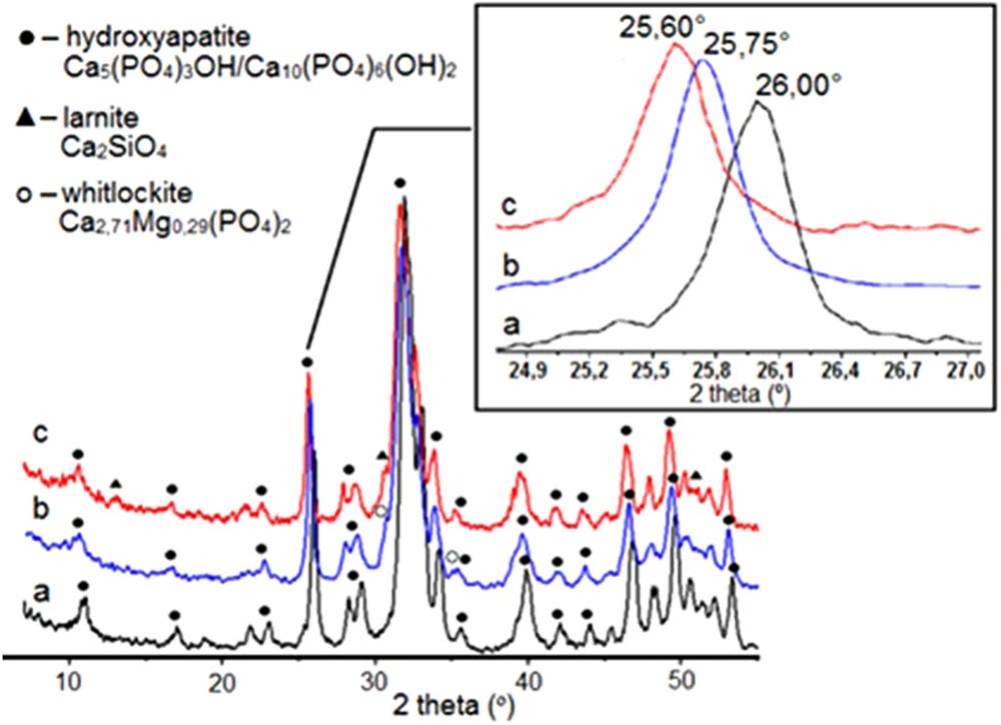
XRD patterns of obtained products: (**a**) HA, (**b**) MgHA, (**c**) SiHA. (●) – HA Ca_5_(PO_4_)_3_OH/Ca_10_(PO_4_)_6_(OH)_2_, (▲) – larnite Са_2_SiO_4_, (**о**) – whitlockite Ca_2,71_Mg_0,29_(PO_4_)_2_.

The XRD patterns in Fig. 1 showed that the main phase of MgHA and SiHA samples was hydroxyapatite with a unit cell of the hexagonal system Ca_5_(PO_4_)_3_OH_hex_. It is known that the lowering of the crystal structure symmetry to some subgroup of the spatial symmetry group of the basic structure usually occurs either due to the displacement of atoms from their particular positions or due to the substitution of some other atoms. Thus one can assume the formation of solid solutions during the preparation of a modified HA, which was confirmed by the shift of the reflections to the region of smaller angles in the XRD patterns (Fig. 1).

Despite the fact that the Mg/Ca pair had more than 30% difference in ionic radii (r_Mg_^2+^ = 0.74 Å; r_Са_^2+^ = 1.04 Å), their isomorphous substitutions are widely known as minerals of the clinopyroxene group (Ca,Mg)SiO_3_, calcite CaCO_3_, magnesite MgCO_3_, garnets, amphiboles, and others. Isomorphous substitutions in the phosphate-silicate system occurred with the formation of solid solutions of the substitution type, because in the crystallochemical aspect a tetrahedral oxygen environment is typical both for phosphorus (V) (r_P_^5+^  = 0,35 Å; r_Si_^4+^  = 0,39 Å) and silicon (IV).

As it follows from the well-known ideas of interactions in isomorphous pairs of Са^2+^/Mg^2+^ and P^5+^/Si^4^, one can assume that the solid solutions formed in the MgHA and SiHA samples belonged to the substitution type.

**Figure 2:**
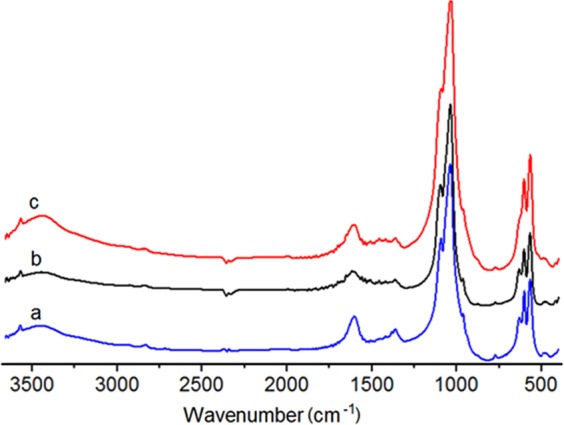
IR spectra of the obtained products: (**a**) HA, (**b**) MgHA *х* = 0.1, and (**c**) SiHA *х* = 0.6.

**Figure 3:**
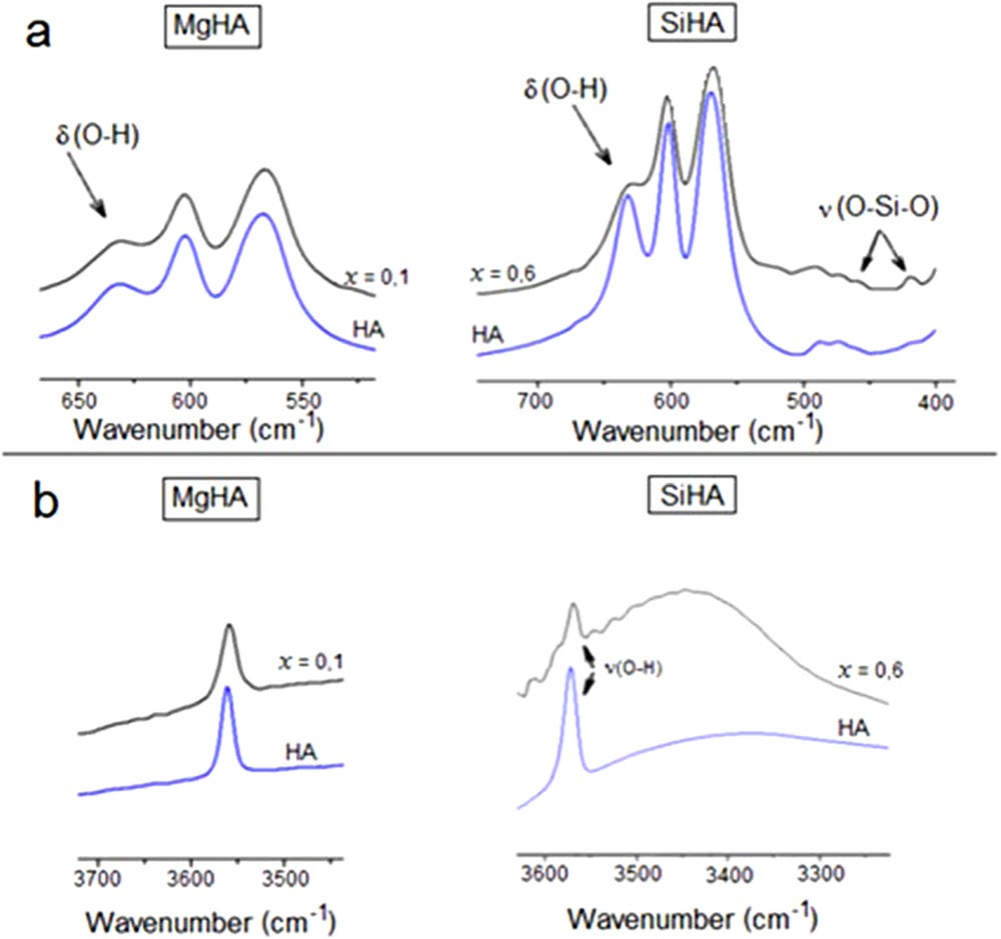
Fragments of IR spectra of unmodified and magnesium- and silicon-modified HA, characterising the vibrations of bonds: (**а**) ν (Р–О) and (**b**) ν (О–Н).

IR spectra of MgHA samples with x = 0.1 and SiHA with x = 0.6 (Figs 2 and 3) indicated the presence of absorption bands characteristic of vibrations of all HA functional groups: blands corresponding to stretching vibrations of OH groups (3570 cm^−1^), valence vibrations (950–1200 cm^−1^), and deformation vibrations (560–610 cm^−1^) of phosphate groups. The decrease in the relative intensity of bands corresponding to deformation vibrations of OH groups at 630 cm^−1^, which occurs in samples of MgHA with different contents of magnesium ions, is probably due to a decrease in the content of hydroxyapatite Ca_5_(PO_4_)_3_OH phase, with simultaneous increase of the content of the whitlockite Ca_2,71_Mg_0,29_(PO_4_)_2_ phase. The decrease in the intensity of the bands for OH groups’ valence (3570 cm^−1^) and deformation (630 cm^−1^) vibrations (Fig. 3) in SiHA samples with different contents of silicate ions infers the partial incorporation of SiO_4_^4−^ ions into the HA structure by the heterovalent substitution mechanism^25^.

### Quantitative composition

**Figure 4:**
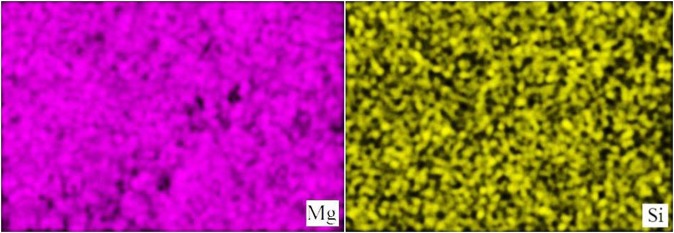
The distribution of elements of Mg and Si on the surface of MgHA and SiHA.

The elemental composition of the modified HA was determined by the results of EMPA (Table 1). The mapping of the surface showed a uniform distribution of modifying ions over the sample surface (Fig. 4).

**Table 1:** Mg and Si content in magnesium-modified and silicon-modified HA.

MgНА			SiНА			НА
ω (Mg), wt.%		(Са + Mg)/P	ω (Si), wt.%		Са/(P + Si)	Са/P
Found	Calculated	1.61	Found	Calculated	1.72	1.66
0.20 ± 0.03	0.24		1.51 ± 0.10	1.69

Slightly lower values of magnesium and silicon contents in MgHA and SiHA powders were because the Mg^2+^ and SiO_4_^4−^ ions partially remained in the mother liquor during the liquid-phase synthesis of the samples.

Quantitation phase by XRD (Table 1) disclosed that MgHA contained the phase of whitlockite (~9 wt.%) and SiHA contained a phase of larnite (~11 wt.%). All samples contained up to 10 wt.% of the phase of hydroxyapatite with monoclinic syngony (Table 2).

**Table 2:** Quantitative assessment of the phase content (wt.%) in unmodified HA, magnesium-modified HA, and silicon-modified HA.

Phase	Syngony	НА	MgНА	SiНА
Ca_5_(PO_4_)_3_OH	Hexagonal	98	85	79
Ca_10_(PO_4_)_6_(OH)_2_	Monoclinic	2	6	10
whitlockite Ca_2,71_Mg_0,29_(PO_4_)_2_	Hexagonal	—	9	—
larnite Са_2_SiO_4_	Hexagonal	—	—	11

A quantitative assessment of the whitlockite and larnite phase contents in MgHA and SiHA samples from XRD data allowed independent estimation of the quantitative content of Mg and Si in these samples (Table 2). The XRD data were in good agreement with the results of EMPA. This was also indicated by the change in the (Ca + Mg)/P ratios in the samples of MgHA and Ca/(P + Si) ratios in the samples of SiHA, which are in stoichiometric HA amounts of 1.67 and remained constant in the case of complete isomorphic replacement of the crystal lattice ions of HA with Mg^2+^ and SiO_4_^4−^ ions. The change in the ratio of these quantities was attributed to the presence of whitlockite and larnite phases.

The volume of elementary hexagonal and monoclinic cells of the phases of hydroxyapatite, calculated by the formulas ${V}_{hex}=(\frac{\sqrt{3}}{2}){a}^{2}$, ${V}_{mon}={\rm{a}}\cdot {\rm{b}}\cdot {\rm{c}}\cdot \,\sin \,{\rm{\beta }}$, whose parameters are given in Table 3. The distortions of the parameters of the elementary cell of the hydroxyapatite phases in the composition of the MgHA and SiHA samples were caused by the incorporation of modifying ions into the crystal lattice of HA. In the case of MgHA, this is due to the lower value of the ionic radius of magnesium relative to calcium; for SiHA, to replacing OH groups with silicates by the heterovalent substitution mechanism of SiO_4_^4−^ ↔ PO_4_^3−^ + OH^−^ for the purpose of charge compensation.

**Table 3:** Phase element parameters HA_hex_ and HA_mono_ as part of the synthesis products of pure, magnesium- and silicon-modified HA.

Sample	HA_hex_			HA_mono_				
	*а* = *b*, Å	*c*, Å	V, Å^3^	а, Å	b, Å	C, Å	β, °	V, Å^3^
HA	9.485	6.937	540.5	9.472	18.911	6.796	119.16	1063
MgHA	9.409	6.873	526.9	9.519	18.748	6.784	118.98	1060
SiHA	9.424	6.883	529.4	9.407	18.808	6.784	118.98	1050

### Microstructure and dispersion

**Figure 5:**
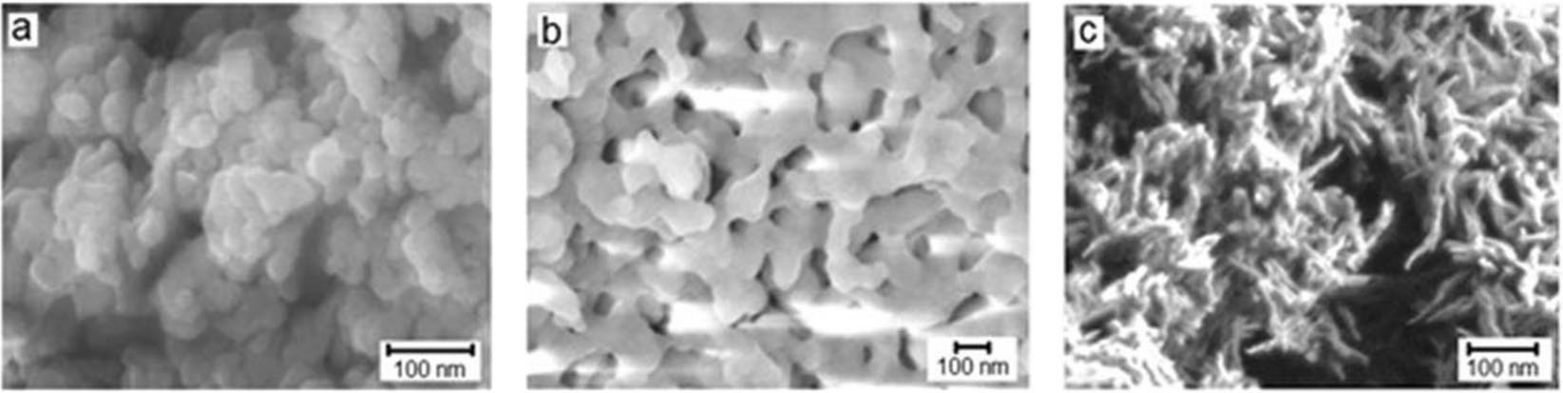
SEM micrographs of the surface of powders (**a**) HA, (**b**) MgHA, and (**c**) SiHA.

The modification of HA with Mg^2+^ and SiO_4_^4−^ resulted in changes of the microstructure, particle sizes, specific surface area and degree of dispersity of the samples. In the photographs obtained on a JEOL-7500FA microscope (Fig. 5), the change of the microstructure of the modified HA depending on the modifying ion is visible. In the case of MgHA, there are no distinct individual particles, in contrast to the literature results^29^; due to the aggregation of particles, an increase in the area of contact occurs. In the case of SiHA, the needle-like particles are ~20 nm diameter and 100 nm length.

**Figure 6:**
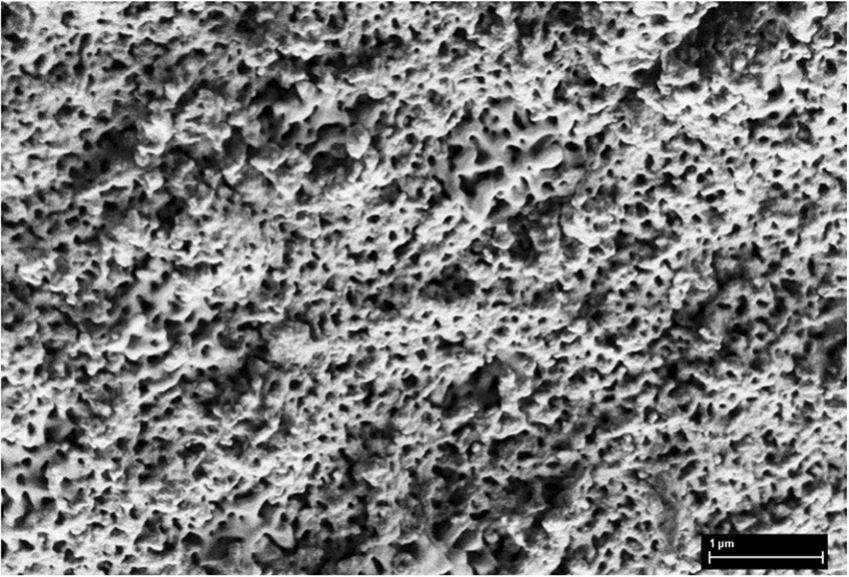
Micrograph of the surface of an MgHA powder.

Note that the surface of MgHA (Fig. 6) contained several regions with different morphologies. It can be assumed that these are different phases, that is, HA and whitlockite.

The density values of powders, determined by the pycnometric method, were (2.56 ± 0.05) g/cm^3^ for MgHA and (2.58 ± 0.07) g/cm^3^ for SiHA allowed one to estimate the particle size of the investigated powders taking into account the results of specific surface area measurements (Table 4).

**Table 4:** The sizes of crystallites (d_Ssp_) particles and the specific surface area (S_sp_) of the obtained products: НА, MgНА, and SiНА.

Microstructure parameter	НА	MgНА	SiНА
S_sp_, m^2^/g	106	74	84
Total pore volume, cm^3^/g	0.40	0.41	0.33
Average pore size, nm	14–20	19–22	13–16
d_Ssp_, nm	22	32	28

According to the specific surface area measurements, an increase in the size of the HA particles after modification was observed in the order HA> SiHA> MgHA. Due to the different morphologies and the tendency of the particles in the modified powders to aggregate, this regularity was not clearly seen in microphotographs (Fig. 5).

### Acid–base properties

**Figure 7:**
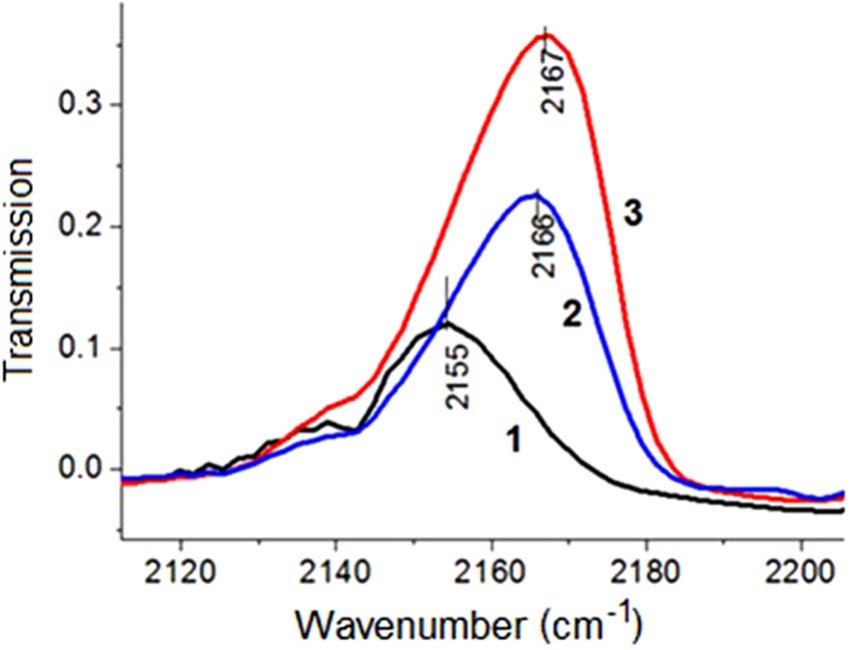
IR spectra of adsorbed СО on the surface of hydroxyapatites at −196 °С: 1 - HA, 2 - MgHA, and 3 – SiHA.

The surface characteristics are important in studying the properties of biomaterials, since they depend on such vital processes as adhesion of proteins, cells, and bioresorbability of materials when they are implanted in the body. As demonstrated by low-temperature adsorption of CO (Fig. 7), the surface of HA, MgHA, and SiHA samples had Lewis acid sites, probably being the coordinatively unsaturated Са^2+^ and Mg^2+^ (in MgHA samples) cations. These sites were characterised by CO oscillation frequencies at 2150–2170 cm^−1^. The strength of Lewis acid sites and their concentrations in the samples of ion-modified HA increased in the order HA < MgHA < SiHA, as evidenced by the shift of CO absorption bands to higher frequencies (Fig. 7) and an increase in their intensity. The increase in the strength of the Lewis acid sites appeared to be due to the higher electronegativity (χ) of magnesium and silicon (χ_Mg_ = 1.31, χ_Si_ = 1.90) relative to calcium (χ_Ca_ = 1.00). The increase in the strength of the Lewis acid sites in the case of SiHA could also be attributed to the presence of vacant orbitals of the 3*d*-subshell in the silicon atom, which acted as lone pair acceptors of CO molecules.

### Solubility and biomimetic properties

The solubility analysis for the samples of modified HA in physiological solution at room temperature (20 °C) and human body temperature (37 °С) is shown in Table 5.

**Table 5:** Solubility of hydroxyapatite powders in physiological solution at рН 7, ω (NaCl) = 0.9%.

Concentration of Ca^2+^ ions in the saturated solution HA with respect to physiological solution		НА	MgНА	SiНА
С_Ca_^2+^·10^3^, mol/l	20 °С	1.59 ± 0.03	2.04 ± 0.07	2.35 ± 0.20
	37 °С	1.74 ± 0.04	2.19 ± 0.12	2.26 ± 0.15

The higher concentration of calcium ions detected in MgHA and SiHA samples in saturated with respect to HA physiological solution was associated with a higher water solubility of whitlockite and calcium silicate phases, respectively, compared to the unmodified HA. An increase of solubility for modified HA had a beneficial effect on the resorbability of materials based on it.

**Figure 8:**
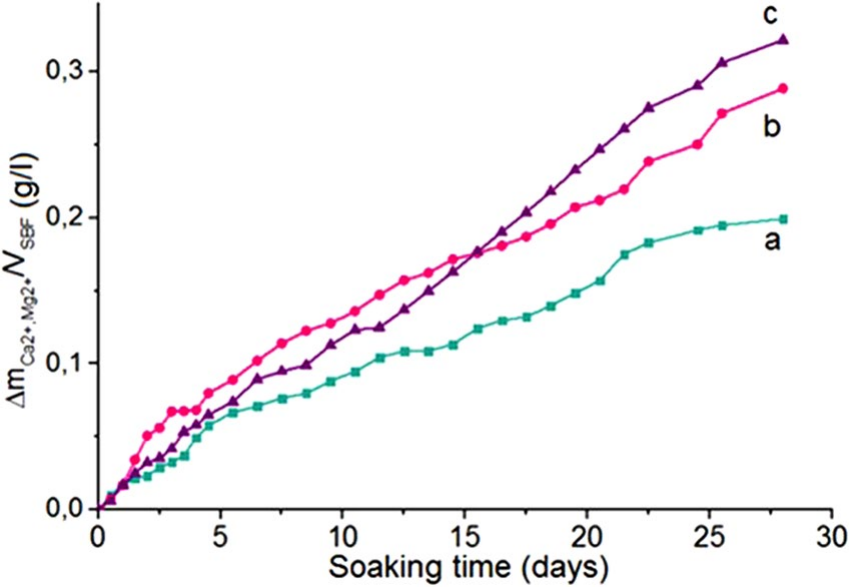
Accumulation curves for Ca^2+^ and Mg^2+^ ions on the surface of tablets places into the SBF solution: (**a**) HA, (**b**)MgHA, and (**c**) SiHA.

**Figure 9:**
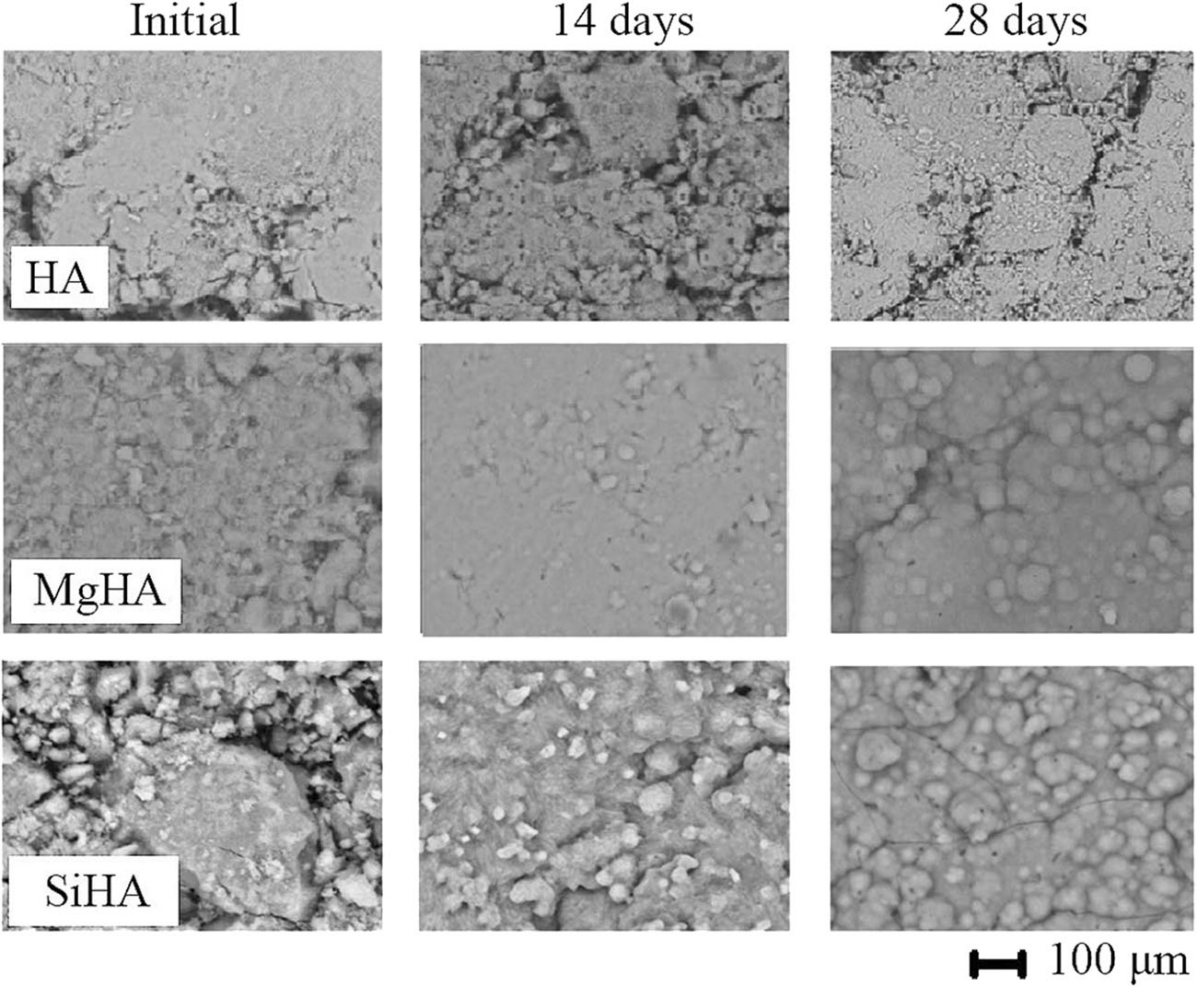
Micrographs of the substrate surface showing the growth dynamics of the calcium–phosphate layer: (**a**) HA, (**b**) MgHA, and (**c**) SiHA.

The biomimetic studies of HA, MgHA, and SiHA samples were carried out in a model SBF solution. From the shape of the kinetic curves (Δ*m*_(Са2+ and Мg2+_), g/l - τ, day) of accumulation of compounds containing calcium and magnesium ions on the substrate surfaces (Figs 8 and 9), one can see that adsorption of Са^2+^ and Мg^2+^ ions from SBF solution was slower on the surface of unmodified HA than on the surfaces of modified HA. This seemed to be due to the increase in the acid properties of the modified HA surface and the appearance of phase boundaries, as a result of which the rate of diffusion and adsorption of counter ions from the SBF solution to the sample increased. Two regions were observed for all curves. The first period lasted about 5 days. The highest rate of ion accumulation Са^2+^ and Мg^2+^ after 5 days took place with the MgHA sample. The highest rate of ion accumulation Са^2+^ and Мg^2+^ after 28 days took place with the SiHA sample. In the period of 5–28 days, the accumulation in HA and MgHA samples was slower, while on the surface of SiHA sample it was slightly faster (Table 6). Silicon modification demonstrated the fastest accumulation rate, and also showed a correlation with the force of coordination-unsaturated centres of the surface. The coefficient of ion accumulation Са^2+^ and Мg^2+^ was calculated by the formula $k=\frac{\varDelta m\,({\rm{Ca}}2+\,{\rm{and}}\,{\rm{Mg}}2+)}{{\rm{\tau }}}$, Δ*m*_(Са2+ and Мg2+)_, g/l – the total change in mass during the time interval τ (days).

**Table 6:** Coefficient of ion accumulation Са^2+^ and Мg^2+^ in HA, MgHA, and SiHA samples.

Sample	*k* (0–5 days)	*k* (6–28 days)
HA	0.388	0.187
MgHA	0.528	0.277
SiHA	0.346	0.362

The formation of CPL on the HA, SiHA, and MgHA substrates (Fig. 8) occurred after 14 days of aging in SBF solution. At day 28 the presence of CPL became apparent and the size of calcium phosphate grains on the surface layer of the newly formed CPL was 2–7 μm.

**Figure 10:**
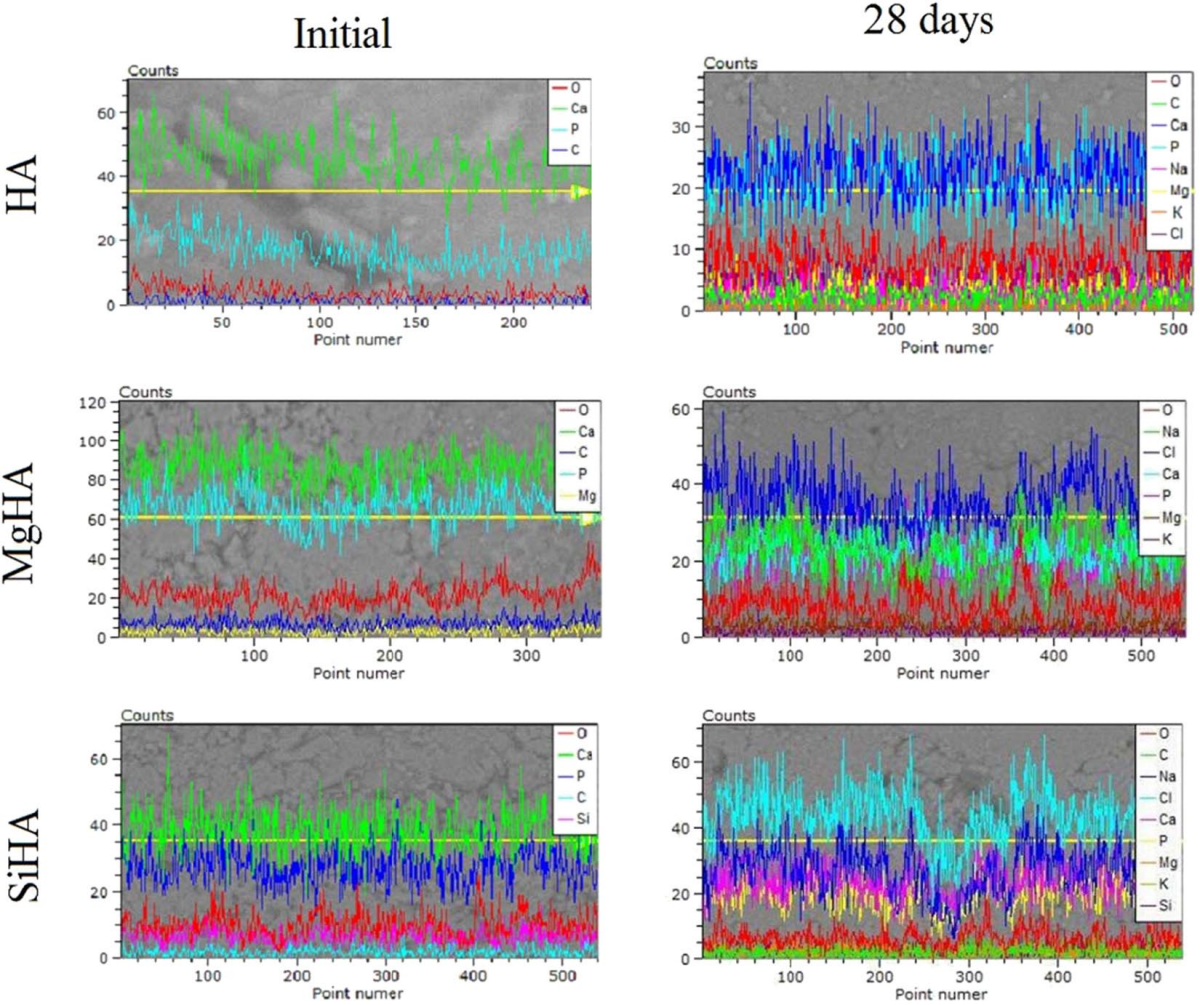
Linear distribution of elements on the surface of the tablets formed on the basis of HA and magnesium- and silicon-modified HA before immersion in the solution (left) and after immersion in the SBF solution for 28 days (right).

After 28 days in the SBF solution, the samples showed an increase in the amount of calcium phosphates on the surface, which is clearly seen in the EPMA (Fig. 10).

It can be assumed that the coordination-unsaturated centres formed acid sites after adsorption of water on the hydroxyapatite surface according to the following mechanism:

$${{\rm{Ca}}}^{2+}+{{\rm{H}}}_{2}{\rm{O}}\to {{\rm{Ca}}}^{2+}\leftarrow :{{\rm{OH}}}_{2}\to {{\rm{CaOH}}}^{+}+{{\rm{H}}}^{+}$$

which acted as fixation centers and further formation of calcium phosphate grains.

## Conclusion

Powders of unmodified HA as well as magnesium- and silicon-modified hydroxyapatites were synthesized by liquid-phase microwave synthesis. The synthesis of HA in the presence of Mg^2+^ and SiO_4_^4−^ ions resulted in the formation of biphasic products. The Mg^2+^ and SiO_4_^4−^ ions were present in the synthesised products both as secondary phases and as part of the HA phase. Whitlockite was found to be present in the magnesium-modified HA (MgHA) and larnite was found to be present in the silicon-modified HA (SiHA); ion substitution for both materials resulted in solid solutions.

In the modified HA powders, an increase in the particle size was observed in comparison to the unmodified HA, and consequently particle size decreased in the order HA < SiHA < MgHA. The Lewis acid sites (Ca, Mg, Si) were present on the surface of the samples of HA, MgHA, and SiHA, and their strength decreased in the order SiHA > MgHA > HA, which was associated with greater electronegativity of magnesium and silicon with respect to calcium. The rate of calcium phosphate layer formation on the surface of material at 37 °C in the model SBF solution depended on the composition of the materials SiHA > MgHA > HA.
